# Autophagy in Hepatic Steatosis: A Structured Review

**DOI:** 10.3389/fcell.2021.657389

**Published:** 2021-04-15

**Authors:** Vitor de Miranda Ramos, Alicia J. Kowaltowski, Pamela A. Kakimoto

**Affiliations:** Departamento de Bioquímica, Instituto de Química, Universidade de São Paulo, São Paulo, Brazil

**Keywords:** liver, steatosis, autophagy, rodent models, review

## Abstract

Steatosis is the accumulation of neutral lipids in the cytoplasm. In the liver, it is associated with overeating and a sedentary lifestyle, but may also be a result of xenobiotic toxicity and genetics. Non-alcoholic fatty liver disease (NAFLD) defines an array of liver conditions varying from simple steatosis to inflammation and fibrosis. Over the last years, autophagic processes have been shown to be directly associated with the development and progression of these conditions. However, the precise role of autophagy in steatosis development is still unclear. Specifically, autophagy is necessary for the regulation of basic metabolism in hepatocytes, such as glycogenolysis and gluconeogenesis, response to insulin and glucagon signaling, and cellular responses to free amino acid contents. Also, genetic knockout models for autophagy-related proteins suggest a critical relationship between autophagy and hepatic lipid metabolism, but some results are still ambiguous. While autophagy may seem necessary to support lipid oxidation in some contexts, other evidence suggests that autophagic activity can lead to lipid accumulation instead. This structured literature review aims to critically discuss, compare, and organize results over the last 10 years regarding rodent steatosis models that measured several autophagy markers, with genetic and pharmacological interventions that may help elucidate the molecular mechanisms involved.

## Introduction

Liver steatosis refers to the accumulation of neutral lipids within organelles called lipid droplets (LDs) located in the hepatocyte cytoplasm. Currently, non-alcoholic fatty liver disease (NAFLD), an umbrella term used to define different conditions in which LDs are present in more than 5% of the hepatocyte and are associated with metabolic diseases (Godoy-Matos et al., 2020) has attracted growing interest. NAFLD is categorized by histological grading of liver biopsies, considering the presence and extension of steatosis, hepatocyte ballooning, and inflammation. Non-alcoholic fatty liver (NAFL) refers to simple steatosis, while non-alcoholic steatohepatitis (NASH) requires hepatocyte ballooning and inflammation (Kleiner et al., 2005; Jahn et al., 2019). Fibrosis may be present in NASH and indicates a more critical scenario that can evolve to irreversible cirrhosis and hepatocellular carcinoma (Loomba and Adams, 2019). There are no approved treatments for NAFLD, and liver transplantation is increasingly necessary for its irreversible consequences (Younossi, 2019).

Notably, NAFLD is widely heterogeneous among patients, and only a small fraction (about 20% of NASH) will develop cirrhosis and liver-derived complications. It is still unclear why. The disease’s pathogenesis was once defined as the result of “two hits”: first lipid accumulation in the hepatocyte, followed by secondary injury caused by inflammation or oxidative stress, for instance (Day and James, 1998). Over the last 20 years other researchers revisited this hypothesis, more consensually proposing that multiple hits, in parallel, may cause NASH (Tilg and Moschen, 2010; Tilg et al., 2020). Mechanistically, the literature consensus is that steatosis can lead to injury, but injury itself, e.g., inflammation, can also lead to steatosis. In 2018, an international expert consensus defined that Metabolic Associated Fatty Liver Disease (MAFLD) is a more appropriate acronym to define steatosis associated with metabolic dysfunction. It considers more universal environmental, metabolic, and genetic factor influences for each patient. This may help to destigmatize patients (due to presumed culpability of “alcoholic” vs. “non-alcoholic”), classify the variants of the disease better, and further improve therapeutic strategies (Eslam et al., 2020).

Indeed, different environmental and genetic factors are known to trigger the development of NAFL and NASH, and mechanisms responsible for the progression of the disease are still being elucidated. At the cellular level, multiple organelles and cellular functions have been found to be impaired. The resulting disorders may be turning point events in NAFLD progression and aggravation, and include endoplasmic reticulum (ER) stress, lysosomal dysfunction, mitochondrial dysfunction, oxidative stress, and impaired autophagy (Lian et al., 2020). Of particular interest, autophagy is a major mechanism controlling liver metabolism, not only as a nutrient stress rescue pathway, but also in physiological responses to fasting-feeding cycles (Ueno and Komatsu, 2017).

There are three types of autophagy: (1) macroautophagy, in which the autophagosome is formed and sequesters cellular components for degradation in lysosomes, (2) microautophagy, in which small parts of cytoplasm are directly sequestered and degraded by endosomes and lysosomes, and (3) chaperone-mediated autophagy, in which proteins containing the KFERQ motif are targeted toward direct degradation in lysosomes. In this review, we will focus on macroautophagy, and call it “autophagy” from now on.

In lipid metabolism, autophagy has been found to regulate both the degradation and formation of LDs (Kwanten et al., 2014). Singh et al. (2009) first characterized the relationship between degradation of lipids and autophagy activation, and coined this process “lipophagy.”. They suggest that basal autophagy regulates triglyceride (TG) content in the liver and is important for lipid mobilization during fasting. On the other hand, Shibata et al. (2009, 2010) showed that microtubule-associated protein 1 light chain 3 (LC3), a crucial autophagy protein, is involved in LD formation. Moreover, autophagy during NAFLD is found to be either active or impaired in many studies using similar models. Thus, the contribution of autophagic activity toward lipid metabolism and, consequently, for steatosis development is still a matter of intense debate in the literature.

In this review, we aimed to evaluate the last 10 years of literature results about steatosis rodent models that measured several autophagy markers, with genetic and pharmacological interventions, in a structured manner. We prepared our tables and focus article lists by searching for “autophagy AND liver AND (nafld OR obesity)” in Scopus and Pubmed, followed by double-blind exclusion in Rayyan QCRI (Ouzzani et al., 2016). We also excluded reviews, experiments not performed in rodents, and papers published in languages other than English. We retrieved all original papers evaluating autophagy markers in steatosis and summarized them by the type of model and results obtained on steatosis and autophagic markers measured in the liver. Our aim was to identify and discuss molecular mechanisms behind the following questions: (1) How is autophagy affected by steatosis? (2) Which molecular pathways of autophagic flux are mainly targeted? (3) Is there a difference in autophagic participation in chronic nutritional overload and fasting-induced steatosis? (4) How can pharmacological interventions and autophagy knockout models help elucidate the interplay between autophagic flux and steatosis?

## Diet-Induced Steatosis Models

### Diets Enriched in Fat

Diets containing more than 30% of energy from fat are considered high-fat diets (HFDs) and models of diet-induced obesity (DIO) and liver steatosis (Jahn et al., 2019). Carbohydrate sources are usually substituted by fat from lard or vegetable oils, and protein is kept around 15–20%. HFDs are widely variable in composition and may be described under other names reflecting quantities of sucrose, fructose, and/or cholesterol, e.g., “high-fat/high-sucrose,” “high-fat/high-cholesterol,” “Western diet,” etc. As a NAFLD model, HFDs containing just excessive fat promote NAFL, but induce NASH only after a long period of feeding (>1 year). Other strategies are used to speed up NASH development, by adding cholesterol and fructose directly to the food, as in the Western Diet, adding fructose and/or sucrose to the water, or using a chemical “disease catalyzer” such as carbon tetrachloride, in low doses (Tsuchida et al., 2018; Boland et al., 2019; Jahn et al., 2019).

We summarized the main diets retrieved from our search (Table 1), but others exist in the literature. A point of concern is that many authors compare HFDs to chow diets, which we and others do not advise (Hariri and Thibault, 2010; Lai et al., 2014; Kakimoto and Kowaltowski, 2016; Preguiça et al., 2020). The source and quantity of ingredients can vary profoundly between chow diets and nutrient-defined diets, compromising metabolic outcome comparisons. The best control diets are purified, containing the same ingredients normalized by total calorie content, changing quantity and not quality, and keeping micronutrients, proteins, and fibers in the same proportions. Notably, manufacturers suggest adequate matching controls for each intervention in open-access databases. In addition to care with control diets, it should be noted that, while these dietary/sedentary models for DIO/steatosis have advantages, they do not necessarily resemble human NAFLD at the molecular level. Each model’s limitations has been thoroughly discussed elsewhere (Friedman et al., 2018; Jahn et al., 2019; Preguiça et al., 2020).

**TABLE 1:** Diets used to induce steatosis and their components.

Name	% fat	% sucrose	% fructose	% added cholesterol
High-fat diet (HFD)	>30 ≤60	≤10	0	≥0 ≤2
High-fat/high-sucrose diet	> 30 ≤60	>10 <45	0	≥0 ≤2
Western diet	∼40	0	>30	≥0 ≤2

**FIGURE 1:**
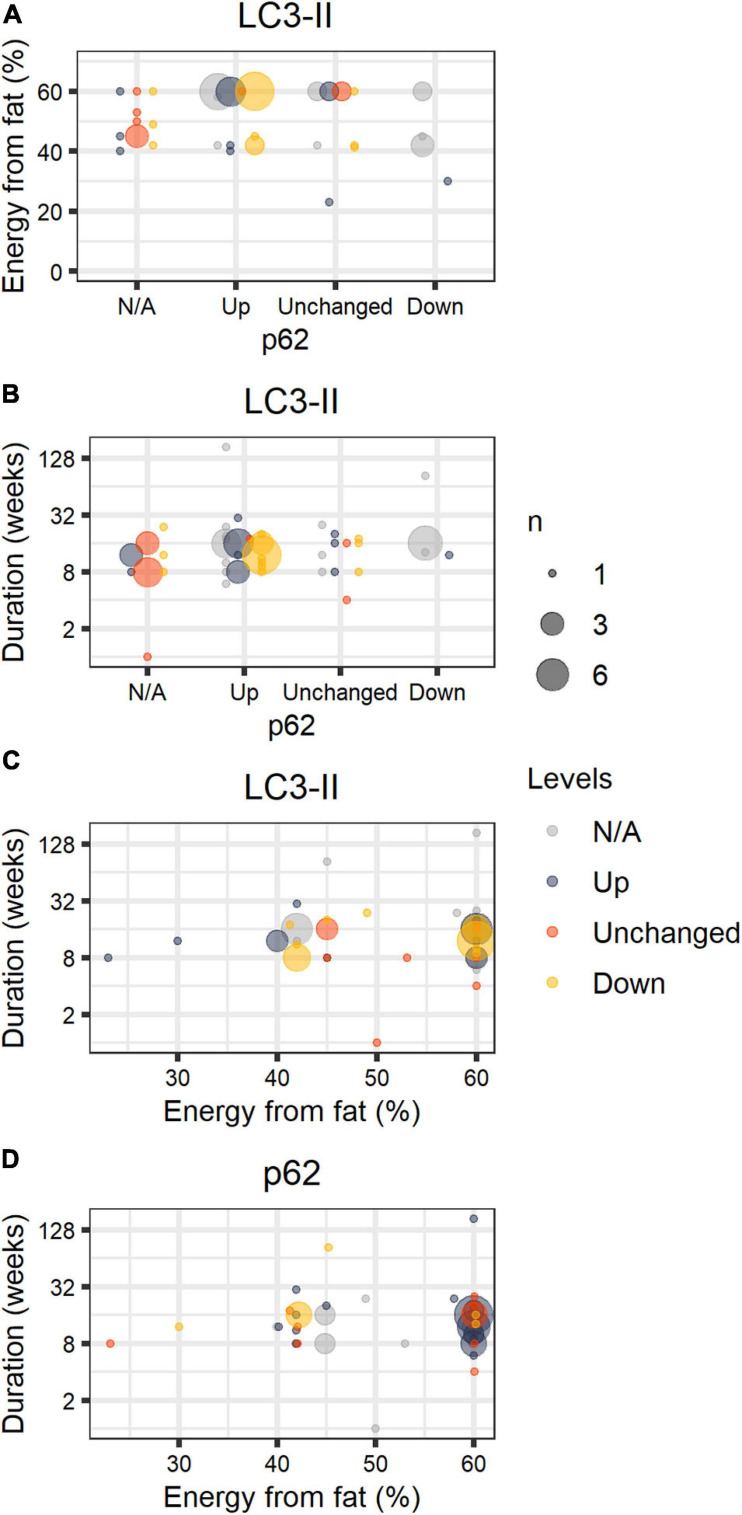
HFD papers. Number of journal articles (*n*) describing diet fat content (in percentage of energy), duration of the intervention, and LC3-II and p62 protein levels in the liver in comparison to control groups (chow or LFD), normalized by a housekeeping protein. LC3-II and p62 were estimated by the authors through SDS-PAGE Western blots and described semi-quantitatively or qualitatively. The size of the circle corresponds to the total number of articles with the same description. Colors refer to LC3-II **(A–C)** or p62 **(D)** qualitative status. [gray = N/A (not available), blue = Up, orange = Unchanged, yellow = Down].

Figure 1 shows compiled information from our search considering the percentage of energy from fat, duration of the dietary intervention, and levels of lipidated LC3 and p62/sequestosome-1 (SQSTM-1), detected by SDS-PAGE and Western blots, compared to animals fed chow or LFD. Both proteins are widely used as monitors of autophagy activation/flux in the literature; LC3-II is the molecular signature of autophagosome formation, and binds to p62, favoring cargo capture for degradation (Pankiv et al., 2007). We highlight that comparisons must be interpreted with caution, as thoroughly discussed by Klionsky et al. (2021).

Excluding papers that involved genetic interventions (Supplementary Table 1), our study compiled 56 papers and 59 comparisons (papers are listed in Supplementary Table 1). We did not include publications in this structured analysis that omitted percentages from fat, duration of the diet, and the status of steatosis. We found that HFD promoted steatosis in virtually all studies, most of which were performed in male C57BL/6 mice fed 60% energy from fat. Animals were fed for 1–184 weeks; 17 ± 22 weeks on average. In general, authors suggest impaired autophagic flux occurs in HFD, accompanied by lipid accumulation. We find that LC3-II levels varied widely even under similar setups, while p62 is mostly accumulated in animals fed 60% energy from fat, and unchanged or reduced when fat content was lower than 60%.

The smaller number of papers using genetically-modified mice and HFD are summarized in Supplementary Table 2. We added information comparing with same genotype mice fed chow or LFD. Out of 14 publications, 9 still developed steatosis. The remaining 5 did not report more lipid accumulation over the control counterpart. Curiously, levels of LC3-II and p62 are mostly unchanged by these interventions.

Studies without genetic interventions (Supplementary Table 1) vary widely in the duration of the HFD-feeding, but autophagic flux seems to be compromised after 6 weeks, when steatosis is already settled. Some experimental designs are useful to find differences in the models and elucidate the mechanism behind autophagic flux alteration. Hsu et al., 2016, followed steatosis, ER stress, autophagy, and apoptosis markers from 2 to 16 weeks of HFD (45% of fat, 20% of sucrose as energy sources). In comparison to chow, lipid accumulation was always identified, while AMPK (adenosine monophosphate-activated protein kinase) phosphorylation increased at 4 and 8 weeks, and fell at 16 weeks. Total LC3-II was increased at 8 weeks and its ratio to LC3-I increased at 16 weeks. PERK (protein kinase R-like endoplasmic reticulum kinase) phosphorylation was reduced at all time points tested, while cleaved caspase 12 was increased only at 2 and 4 weeks, and PARP (poly ADP-ribose polymerase) at 8 weeks (Hsu et al., 2016). While other markers were not assessed, from this data it seems lipid accumulation and unfolded protein response (UPR) activation occur first, and autophagy flux is probably increased at 8 weeks, after AMPK activation. Similar findings were observed in the spontaneous OVE26 diabetic mice, but over 5–8 months (Zhang Q. et al., 2015). Accordingly, high-fat fed rats submitted to sleeve gastrectomy showed significant TG reduction, and lower markers of ER stress and UPR activation, accompanied by increased β-oxidation, AMPK and autophagic activation (Ezquerro et al., 2016, 2020). Similar changes were found using roux-en-Y gastric bypass interventions (He et al., 2015; Ma et al., 2020). ER stress and UPR activation are common findings in the steatotic liver of rodents and humans, and many targets have been suggested as possible points of intervention for NAFLD treatment (Lebeaupin et al., 2018).

To evaluate the effect of type (not quantity) of fats on liver steatosis and autophagy markers, Wang M.-E. et al. (2017) formulated a diet analogous to the Research Diets D12492 (60% energy from fat, containing soybean oil and lard as fat sources), but substituting about half of the lard for coconut oil. This yields a higher proportion of medium-chain fatty acids (MCFA) in comparison to long-chain fatty acids (LCFA) (Wang M.-E. et al., 2017). The MCFA-enriched diet promoted similar results to LCFA regarding body weight gain, increased fasting glycemia, and liver steatosis, but to a lesser extent. Animals showed reduced staining with Sirius Red and collagen, suggesting less fibrosis in the MCFA groups. While both diets increased the content of LC3-II, only the LCFA-enriched diet accumulated p62 and increased ER stress markers. The authors conclude that MCFA may alleviate autophagic flux impairment produced by HFD. Consistently, markers of lipotoxicity-induced autophagy impairment in *in vitro* studies could be modulated by increasing the content of MCFA to LCFA in the lipid mix. This may be associated with LCFA-induced expression of RUBICON, a negative regulator of autophagosome fusion to lysosomes, described as a possible regulatory node of autophagy impairment and steatosis induced by HFD (Tanaka et al., 2016).

### Methionine-Choline Deficient Diets

The methionine-choline deficient (MCD) diet is another popular model to quickly induce NASH in rodents (Santhekadur et al., 2018). The rationale is that choline deficiency compromises the export of lipids by the liver, while increasing uptake, and thus promoting accumulation in the cytoplasm (Rinella et al., 2008). MCD diets are usually enriched in sucrose and can vary in the percentage of energy from fat (10–60%). After about 4 weeks of feeding, animals develop steatosis, inflammation, fibrosis, and extensive liver damage. Although it promotes hepatic histology similar to human NASH, the model is dissimilar to most human disease presentations, as animals lose weight and do not become insulin resistant (Friedman et al., 2018). Machado et al. (2015) described that 8 weeks of MCD diet, compared to 16-week Western Diet, promoted severe liver damage, inflammation and fibrosis, but accumulated fewer triglycerides. On the other hand, Western Diet promoted weight gain, more extensive triglyceride accumulation, insulin resistance, and dyslipidemia. The authors suggest that the MCD diet is a reasonable model for liver injury and evolution of NASH, but the Western Diet is more appropriate to study NAFLD and its consequences for other tissues. Since human NAFLD is slowly progressive and will evolve to NASH only in a minority of patients, the MCD diet remains a harsh, but complementary, study model.

We summarized all papers in our search using the MCD diet as a NASH model that evaluated steatosis, LC3-II and/or p62, and compared it to an LFD or chow diet (Table 2). The time course of MCD diet feeding varied from 1.5 to 10 weeks. Most of the studies, except one in male BALB/c mice, were performed in male C57BL/6 mice. Consistently, the MCD diet promoted steatosis and p62 accumulation in all studies. LC3-II was unchanged (1 out of 18 comparisons), increased (12/18), or decreased (5/18). Most authors concluded that autophagic flux is compromised in MCD diet-fed animals. Notably, Wang et al. (2018) found LC3-II and p62 levels increased at 3-weeks of MCD in both C57BL/6 and obese *db/db* mice. HFD promoted similar results at 12 weeks. The authors suggest, by combining many animal and cell lineage models of steatosis, that autophagic flux impairment is a typical result of deficient lysosomal acidification induced by ER stress and asparagine accumulation. The same group described impaired autophagolysosome formation and acidification, mediated by upregulation of the C-X-C motif chemokine 10, using HFHC and MCD as steatosis models (Zhang et al., 2017). Similarly, Tang et al. (2020) promoted steatosis using HFD, the MCD diet, and cell models, and found decreased autophagic flux due to deficient autophagolysosome formation, which the authors ascribe to increased synthesis of osteopontin (OPN). The neutralization of OPN restored autophagic flux and reduced triglyceride accumulation.

**TABLE 2:** MCD model.

References	Strain	Duration of MCD (weeks),% energy from fat, catalog	LC3-II/housekeeping	p62/housekeeping
			
			(vs. Chow or LFD)	
Ji et al., 2015	C57BL/6	4	Up	Up
Machado et al., 2015	C57BL/6	8, 20%, MP Biomedicals, #960439	Down	N/A
Xie et al., 2015	BALB/c	1.5	Up	N/A
Chen et al., 2016	C57BL/6	10	Down	Up
Zhang X. et al., 2016	C57BL/6	4	Up	Up
Zhang X. et al., 2016	C57BL/6 CXCR3 KO	4	Up	Up
Zhang et al., 2017	C57BL/6	4	Up	Up
Lee et al., 2018	C57BL/6	4, 30%	Up	Up
Wang et al., 2018	C57BL/6	3	Up	Up
Wang et al., 2018	*db/db*	3	Up	Up
Cruces-Sande et al., 2018	C57BL/6	4, 10%, Envigo, TD. 90262	Unchanged	Up
Zheng Y. et al., 2018	C57BL/6	3, 10%	Up	Unchanged
Zeng et al., 2018	C57BL/6	5	Down	Up
Wu C. et al., 2018	C57BL/6	4	Down	Up
Veskovic et al., 2019	C57BL/6	6	Up	Up
Chen et al., 2019	*db/db*	4	Up	Up
Li et al., 2019a	C57BL/6	4, 21%, Research Diets, A02082002B	Down	Up
Tang et al., 2020	C57BL/6	8	Up	Up

*Articles using MCD as a NASH model, describing intervention duration, liver steatosis, LC3-II and p62 protein in the liver in comparison to control groups (chow or LFD), normalized by a housekeeping protein. LC3-II and p62 were obtained by the authors through SDS-PAGE Western blots and described semi-quantitatively or qualitatively. All experiments observed increases lipid accumulation in the liver. All animals were male mice (N/A, not available).*

ER stress is commonly observed in lipid overload/lipotoxicity models, and many authors suggest it is the upstream signal of reduced autophagic flux (González-Rodríguez et al., 2014). Interestingly, Hernández-Alvarez et al. (2019) recently described that the mitofusin-2 (Mfn2) binds to phosphatidylserine and is responsible for phospholipid shuttling between ER and mitochondria to produce phosphatidylethanolamine and phosphatidylcholine. Human NASH patients and MCD- and HFD-fed mice have reduced Mfn2 content and aberrant lipid metabolism. Preventing ER stress normalized inflammation and fibrosis markers, but not lipid metabolism (Hernández-Alvarez et al., 2019). While reduced Mfn2 has been strongly associated with impaired autophagic flux in many cell types and tissues (Muñoz et al., 2013; Sebastián et al., 2016), cellular lipid composition and mitochondrial phosphatidylethanolamine synthesis are known to be essential for full autophagic activation (Koga et al., 2010; Thomas et al., 2018). Since phosphatidylethanolamine is incorporated into LC3-I by ATG3 and ATG7 (“ATG” denotes autophagy related proteins), reduced availability in NASH patients may also be a player in impaired autophagic flux.

From these studies comparing many steatosis models, we suggest that steatosis and autophagy flux impairment in the liver may be dissociated from systemic glucose homeostasis and adiposity, and converge as a cellular responses to lipid accumulation *per se*.

### Leptin and Leptin Receptor Deficient Mice

Leptin (*ob/ob*) and leptin receptor (*db/db*) deficient mice are obesity models with spontaneous mutations resulting in lack of leptin signaling. These animals are hyperphagic and have severe body weight gain at early ages (Wang B. et al., 2014). An interesting aspect when comparing *ob/ob* and *db/db* animals to dietary models is that, usually, hyperphagia models are fed with chow diets, containing most of the energy from carbohydrates, and not fat. Obesity is thus the result of hypercaloric ingestion and lack of leptin signaling. The animals spontaneously develop hyperinsulinemia, dyslipidemia, and liver steatosis. A critique of these models is that obese humans become hyperleptinemic and typically leptin-resistant over time (Wang B. et al., 2014).

In Table 3, we summarized our search for papers that evaluated steatosis, lipidated LC3 and/or p62 concomitantly and compared WT mice and *ob/ob* or *db/db* mice as a NAFLD model. All mice used were male from 7 to 22 weeks of age and developed steatosis with chow diets. Like the other obesity and NAFLD models, p62 is accumulated in all papers that measured it (5/5). LC3-II is increased in half the publications, while the other half is decreased (3/6). Overall, autophagic flux is impaired.

**TABLE 3:** Leptin and leptin-receptor deficient mice.

References	Genetics	Final age (weeks)	LC3-II/housekeeping	p62/housekeeping
			
			(vs. WT)	
Liu et al., 2015	*ob/ob*	14	Up	N/A
Hong et al., 2018	*ob/ob*	14	Down	Up
Zheng W. et al., 2018	*ob/ob*	12	Down	N/A
Yang et al., 2020	*ob/ob*	9	Up	Up
Kim et al., 2016	*db/db*	22	N/A	Up
Huang et al., 2017	*db/db*	10	Down	Up
Matsumoto et al., 2019	*db/db*	7	Up	Up

*Articles using leptin- or leptin receptor-deficient mice, describing intervention duration, and LC3-II and p62 protein in the liver in comparison to control groups (WT mice), normalized by a housekeeping protein. LC3-II and p62 were obtained by the authors through SDS-PAGE Western blots and described semi-quantitatively or qualitatively. All experiments observed increases in lipid accumulation in the liver. All animals were male mice (N/A, not available).*

Two of these studies found that increased sirtuin 1 (SIRT1) expression could restore autophagic flux in *db/db* and *ob/ob* mice (Huang et al., 2017; Hong et al., 2018). Both models have decreased SIRT1 protein levels compared to WT. Through different pharmacological interventions, i.e., ginsenoside Rb2, erythropoietin, or resveratrol, authors found that AMPK activation and increased levels of SIRT1 both increase autophagic flux and reduce steatosis. In another study, *ob/ob* mice under calorie restriction or metformin treatment showed increased SIRT1 levels and autophagic flux, as well as reduced steatosis in comparison to *ad libitum* fed animals. *In vitro* experiments suggest a mechanism independent of AMPK of SIRT1-dependent autophagy activation for metformin, which remains to be clarified (Song et al., 2015).

Adenosine monophosphate-activated protein kinase signaling is a central hub in the liver, controlling glycogen and lipid metabolism (González et al., 2020). Its activation counteracts mTOR (mechanistic target of rapamycin kinase) signaling by increasing autophagic flux through the phosphorylation of ULK1 (unc-51 like autophagy activating kinase 1) in Ser317 e Ser777 (Kim et al., 2011), while SIRT1 is known to deacetylate many autophagy proteins, and to be a downstream target of adipose tissue triglyceride lipase (ATGL) induction of lipophagy in the liver (Lee et al., 2008; Sathyanarayan et al., 2017). Furthermore, melatonin and berberine effects on autophagy activation and reduced steatosis were also observed to be SIRT1-dependent in mice under HFD with intact leptin signaling, as confirmed in liver-specific knockouts (Sun et al., 2018; Stacchiotti et al., 2019). Resveratrol is defined as a SIRT1 activator and is described by many authors to increase autophagic flux and alleviate steatosis in many rodent models, including HFD, MCD, and genetic interventions thought to relieve ER stress and inflammation (Li et al., 2014; Ji et al., 2015; Zhang Y. et al., 2015; Ding et al., 2017; Milton-Laskibar et al., 2018). The similar contributions of SIRT1 activation in different models suggest a common point of intervention in steatosis development and subsequent resolution. However, some clinical trials did not observe significant reduction of steatosis upon 12 weeks of treatment (Kantartzis et al., 2018; Farzin et al., 2020), a finding that is still under debate regarding the effects of overdosage and treatment length (Fogacci et al., 2018).

### Mechanisms of Autophagy Impairment in NAFLD

Why NAFLD models lead to impaired autophagy is still debated. Several mechanisms leading to dysfunction of late steps in the autophagic process, such as decreased lysosomal function and autophagosome-lysosome fusion, have been proposed. Interestingly, different literature results suggest that these steps may be impaired as a consequence of ER-stress in NAFLD models. Wang et al. (2018) demonstrated that cellular responses to ER stress promote increased levels of asparagine, leading to decreased lysosome acidification in animals fed MCD or HFHC diets as well as hepatocyte cultures treated with MCD. Moreover, *in vitro* studies by Miyagawa et al. (2016) demonstrated that palmitate-induced ER-stress supports impaired autophagosome-lysosome fusion in hepatic cells, as this was attenuated by co-treatment with chemical chaperone. Curiously, lysosome acidification and function were not altered by palmitate. These studies may suggest that, although all NAFLD models culminate in autophagic impairment, specific mechanisms could be favored under each condition. This has also been suggested by other authors based on different responses of autophagy markers comparing HFD with high sucrose and HFHS diets (Simoes et al., 2020). In addition to these evidences, the UPR and autophagy have recently been shown to share common regulatory pathways. The spliced form of XBP1 (sXBP1), a transcription factor necessary for UPR activation, binds to the promoter region and activates the expression of transcription factor EB (TFEB). This interaction is reduced in steatotic hepatic tissue (Zhang et al., 2020). TFEB is the main transcription factor that binds to the CLEAR regulatory motif and promotes expression of an array of lysosomal and autophagic genes (Napolitano and Ballabio, 2016). Thus, downregulation of TFEB expression may not only lead to decreased lysosome biogenesis and activity, but also affect overall autophagy activation. Along with ER-stress-derived mechanisms, obesogenic diets were shown to promote iNOS localization at the lysosome surface, causing nitric oxide stress, impaired lysosomal function and decreased TFEB activation (Qian et al., 2019). Finally, autophagosome-lysosome fusion may also be suppressed by RUBICON, a BECLIN-1 interacting protein, which was shown to be upregulated in the hepatic tissue of mice fed HFD, and to contribute toward NAFLD progression (Matsunaga et al., 2009; Zhong et al., 2009; Tanaka et al., 2016).

Changes in AMPK and mTORC1 activity are also suggested to mediate impaired autophagy during NAFLD. Recent findings suggest that upregulation of CD36, a protein that facilitates fatty acid uptake in hepatocytes, leads to inhibition of autophagy initiation in fatty livers, through the AMPK/ULK1/BECLIN-1 pathway (Li et al., 2019b; Samovski and Abumrad, 2019). In fact, liver-specific AMPK activation was shown to counteract steatosis development during obesity by different mechanisms, including autophagy activation (Garcia et al., 2019). Bariatric surgery in rodents, for instance, improves steatosis while increasing AMPK and decreasing mTORC1 activation (Ezquerro et al., 2016; Ma et al., 2020). Moreover, increased cytosolic acetyl-CoA derived from peroxisomal β-oxidation present in the liver of HFD fed animals was shown to promote mTOR localization at the lysosome surface, increasing ULK1 phosphorylation by mTORC1 and inhibiting autophagy (He et al., 2020). Although AMPK and mTORC1 are known regulators of autophagy initiation by activation/inhibition of the ULK1/2 complex, both are important modulators of lysosome function, and, therefore, of late steps of the autophagic process (reviewed by Carroll and Dunlop, 2017). For instance, activation of mTORC1 at the lysosome surface is known to phosphorylate TFEB, promoting its cytosolic localization. In contrast, decreased lysosome-derived amino acids, promoted either by nutrient deprivation or by lysosomal dysfunction, can inhibit mTORC1 activity, causing TFEB dephosphorylation and translocation to the nucleus. Conversely, TFEB activation can upregulate lysosomal activity and restore mTORC1 activation (Napolitano and Ballabio, 2016; Zhang et al., 2018). This feedback signaling is known to oscillate in the hepatic tissue and is directly related to steatosis development, while disruptions in favor of mTORC1 activation can exacerbate NAFLD progression (Zhang et al., 2018). mTORC1 activation in NAFLD models may also inhibit autophagosome-lysosome fusion by inactivating PACER protein, a mediator of autolysosome formation (Cheng et al., 2019). Importantly, mTOR activity can also be regulated by AMPK status. Even though AMPK activity can modulate lysosome function by regulating TFEB activation, V-ATPase activity, and calcium transport in different physiological or pathological contexts (McGuire and Forgac, 2018; Bonam et al., 2019; Fernandez-Mosquera et al., 2019; Deus et al., 2020), to the best of our knowledge the participation of similar mechanisms in NAFLD models has not yet been further explored.

Chronic lipid exposure can also lead to altered Ca^2+^ signaling in different tissues, promoting dysregulation of metabolic signals during obesity (Fu et al., 2011; Guney et al., 2020). Increased cytoplasmic Ca^2+^ concentration, decreased ER Ca^2+^ levels and store-operated-calcium-entry (SOCE) are reported in steatotic hepatocytes and are known to contribute toward NAFLD progression (Arruda et al., 2017; Ali et al., 2019). Calcium is an important regulator of autophagic activity (Filippi-Chiela et al., 2016; Bootman et al., 2018). In fact, treatment of obese mice with calcium channel blockers rescued abnormal cytoplasmatic Ca^2+^ levels in hepatocytes and restored impaired autophagosome-lysosome fusion (Park et al., 2014). Additionally, decreased levels of ATG7 protein in steatotic livers are shown to be related to calpain proteolytic activity (Yang et al., 2010). Together, these results indicate that altered Ca^2+^ signaling can affect different steps of autophagy regulation during NAFLD progression and may represent an important signaling hub in this context.

Finally, altered lipid availability during obesity is also thought to contribute toward autophagy impairment. To that end, Koga et al. (2010) demonstrated that *in vitro* and *in vivo* lipid overload can impair autophagosome-lysosome fusion due to altered lipid composition in both membranes. Phospholipid availability is also known to modulate autophagosome formation (reviewed by Hsu and Shi, 2017; Soto-Avellaneda and Morrison, 2020), which may be a contributing factor toward decreased autophagic activity during NAFLD.

**FIGURE 2:**
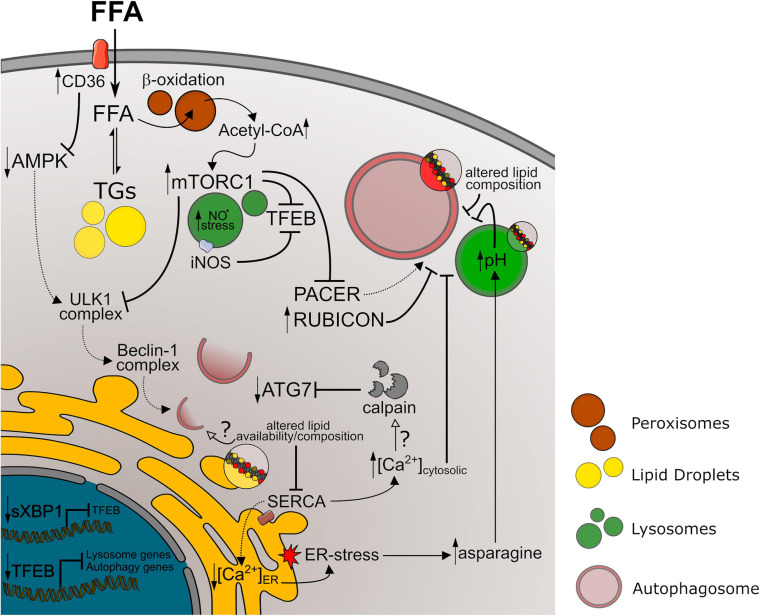
Autophagy impairment in NAFLD models. References to the mechanism represented above are discussed in Section “Mechanisms of Autophagy Impairment in NAFLD” of this manuscript. Longer arrows indicate activation mechanisms, while doted lines or full lines indicate downregulated or upregulated pathways, respectively. Crossed lines indicate inhibitory mechanisms. Small arrows pointing up or down indicate increased or decreased levels/activity, respectively. Unfilled arrows along with question marks indicate hypothetical mechanisms. FFA, free fat acids; TGs, triglycerides; NO^⋅^, nitric oxide; ER, endoplasmic reticulum.

In general terms, dysfunction at the later steps of autophagy pathways leads to accumulation of autophagic vesicles and, therefore, of lipidated LC3. Conversely, decreased autophagy initiation or impaired autophagosome formation leads to lower autophagosome content and decreased LC3-II. In our summary of autophagic markers in NAFLD models, we observed a high variability of LC3-II levels between studies, mostly found either increased or decreased. This corroborates evidence showing that impairment of autophagic activity can occur at different steps of autophagy regulation during NAFLD development. A summary of mechanisms discussed here is represented in Figure 2.

## Autophagy Machinery Interventions

### Autophagic Pharmacological Interventions

In this section we analyzed all articles from our search that evaluated the effects of known pharmacological modulators of autophagy on hepatic steatosis. The results are summarized in Table 4. Data is divided according to experimental design, separating pharmacological interventions administered during diet feeding, at the end of the diet, or after. Overall, we analyzed 27 experiments from 17 different studies in rodent models of diet-induced hepatic steatosis that administered rapamycin as an autophagy activator or chloroquine and 3-methyladenine (3-MA) as inhibitors. Importantly, some experiments evaluated the effects of autophagic modulators alone or in combination with other interventions (named co-interventions). In summary, 18 experiments (from 14 studies) showed a negative correlation between autophagy and steatosis levels, meaning that steatosis was up- or downregulated in the presence of an autophagy inhibitor or activator, respectively. Eight experiments did not present changes in steatosis levels, and one showed a positive correlation, meaning an increase in steatosis after autophagy activation.

**TABLE 4:** Pharmacological intervention on autophagy.

	Animal	Diet (fat concentration, duration)	Pharmacological intervention	Time point of intervention	Duration of intervention	Co-intervention	Autophagy modulation (up/down)	Steatosis outcome (up/down)	Correlation (+/0/−)
Chang et al., 2009b	KK/HlJ	HFD (67%, 10 weeks)	Rapamycin	During diet	Last 6 weeks	No	Up	Down	−
Wang C. et al., 2014	C57BL/6J	HFD (60%, 14 weeks)	Rapamycin	During diet	14 weeks	No	Up	Down	−
Zhang F. et al., 2016	C57BL/6J	HFD (60%, 12 weeks)	Rapamycin	During diet	12 weeks	BCAA	Up	Up	+
Chen et al., 2016	C57BL/6J	MCD (10 weeks)	Rapamycin	During diet	10 weeks	No	Up	Down	−
Ren et al., 2019	C57BL/6	HFD (not described, 6 weeks)	Rapamycin	During diet	6 weeks	Hypoxia	Up	Down	−
Lu et al., 2020	C57BL/6J, male and females	ApoEKO + HFD (not described, 8 weeks)	Rapamycin	During diet	8 weeks	No	Up	Down	−
Zhao et al., 2020	C57BL/6J	HFD (60%, 13 weeks)	Rapamycin	During diet	Last 8 weeks	No	Up	Down	−
Chen et al., 2016	C57BL/6J	MCD (10 weeks)	Chloroquine	During diet	10 weeks	No	Down	Up	−
Zeng et al., 2018	C57BL/6	MCD (5 weeks)	Chloroquine	During diet	5 weeks	Acetylshikonin	Down	Up	−
Ren et al., 2019	C57BL/6	HFD (not described, 6 weeks)	3-MA	During diet	6 weeks	Hypoxia	Down	Up	−
Lin et al., 2013	C57BL/6	HFD (60% calories, 12 weeks)	Rapamycin	End of diet	Acute – before sacrifice	No	Up	Down	−
Lin et al., 2013	C57BL/6	HFD (60% calories, 12 weeks)	Chloroquine	End of diet	Acute – before sacrifice	No	Down	Up	−
Zhang et al., 2018	C57BL/6J, male and female	HFD (60%, 3 weeks)	Chloroquine	End of diet	Acute – before sacrifice	No	Down	Unchanged	0
Zhang et al., 2018	C57BL/6J, male and female	HFD (60%, 10 weeks)	Chloroquine	End of diet	Acute – before sacrifice	No	Down	Up	−
Zhang et al., 2018	C57BL/6J, male and female	HFD (60%, 16 weeks)	Chloroquine	End of diet	Acute – before sacrifice	No	Down	Unchanged	0
Tong et al., 2016	*db/db*	Regular chow	Chloroquine	Not applied	3 days	No	Down	Unchanged	0
Tong et al., 2016	*db/db*	Regular chow	Chloroquine	Not applied	3 days	GW501516	Down	Up	−
Zhu et al., 2020	C57BL/6J	HFD (60%, 12 weeks)	3-MA	End of diet	Last 2 weeks	Nrg4	Down	Up	−
Chang et al., 2009a	C57BL/6J	HFD (67%, 20 week)	Rapamycin	After diet	16 weeks	No	Up	Down	−
Zhou and Ye, 2018	Sprague-Dawley	HFD (45%, 8 week) + STZ	Rapamycin	After diet	12 weeks	No	Up	Down	−
Tu et al., 2020	Sprague-Dawley	HFD (60%, 6 weeks)	Rapamycin	After diet	2 weeks	No	Up	Unchanged	0
Sun et al., 2015	C57BL/6	HFD (not described, 8 weeks)	Chloroquine	After diet	6 weeks	NaHS	Down	Up	−
He et al., 2016a	C57BL/6	HFD (60%, 12 weeks)	Chloroquine	After diet	6 weeks	No	Down	Unchanged	0
He et al., 2016a	C57BL/6	HFD (60%, 12 weeks)	Chloroquine	After diet	6 weeks	Berberine chloride	Down	Unchanged	0
He et al., 2016b	C57BL/6	HFD (60%, 12 weeks)	Chloroquine	After diet	12 weeks	No	Down	Unchanged	0
He et al., 2016b	C57BL/6	HFD (60%, 12 weeks)	Chloroquine	After diet	12 weeks	Liraglutide	Down	Unchanged	0
Zhou and Ye, 2018	Sprague-Dawley	HFD (45%, 8 week) + STZ	3-MA	After diet	12 weeks	No	Down	Up	−

*Articles using pharmacological interventions in the autophagy process in comparison to control groups (vehicle), normalized by a housekeeping protein. LC3-II and p62 were measured by the authors through SDS-PAGE Western blots and described semi-quantitatively or qualitatively. All but two studies used male animals. Positive correlation = steatosis is up, and autophagy is up OR steatosis is down, and autophagy is down; negative correlation = steatosis is up, and autophagy is down OR steatosis is down, and autophagy is up.*

With the exception of one study, all experiments that chronically administered rapamycin to animals during the period of diet feeding showed an improvement in steatosis outcome, suggesting that autophagy activation during the development of NAFLD counteracts TG accumulation in hepatocytes. Of note, the experiment showing a positive correlation between rapamycin administration and steatosis was performed with a co-intervention of branched chain amino acids (BCAA) to mice fed a HFD, which was shown by the authors to severely increase liver injury and to reduce hepatic TG levels, but to increase free fatty acids (FFA) and lipotoxicity; rapamycin reversed these effects (Zhang F. et al., 2016). In agreement with the protective role of autophagy, chronic chloroquine or 3-MA administration with HFD increased steatosis levels compared to HFD alone, or even inhibited the protective effect of other co-interventions.

An interesting study by Zhang et al. (2018) evaluated the effects of acute chloroquine administration to mice before analyzing hepatic TGs and LDs at different time points of HFD feeding. They showed that autophagy inhibition by chloroquine only increased steatosis levels after 10 weeks of HF feeding, without significant effects earlier (3 weeks) or later (16 weeks). These results suggest that autophagic degradation of LDs by lipophagy may not be essential at the initial periods of NAFLD development, but participation may increase with time. Also, decreased lipophagic activity after 16 weeks is probably associated with impaired autophagic flux, which is more evident at this time point compared to 10 weeks. This time course is in agreement with the study from Yang et al. (2010) showing that ATG7 levels are unchanged after 7 weeks of HFD, but decreased after 16 weeks, and virtually absent after 22 weeks.

When evaluating the effects of rapamycin as a therapeutic intervention after HFD feeding, 2 out of 3 studies reported decreased hepatic steatosis, while one did not observe any changes. The main difference that we could observe between these studies was that both reports with an improved outcome administered rapamycin for at least 12 weeks after the diet, while the study without effects only submitted the animals to 2 weeks of treatment. It is therefore possible that therapeutic effects of rapamycin on hepatic steatosis require longer treatment periods. However, we cannot exclude the fact that other experimental differences between the studies may also contribute toward divergent outcomes. In this context, results with autophagy inhibitors administered after HFD feeding were controversial. Two different studies from the same group that administered chloroquine for 6 or 12 weeks after initial 12 weeks of HFD feeding did not observe any changes in steatosis levels (He et al., 2016a, b). In contrast, chronic exposure of 3-MA to rats after 8 weeks of HFD feeding lead to increased steatotic outcome (Zhou and Ye, 2018). Also, chloroquine administration to mice fed HFD for the same time period reversed the protective effects of the co-intervention (Sun et al., 2015). While literature data seems inconclusive, it is possible that inhibition of autophagy after NAFLD development may only upregulate steatosis if autophagic activity is not already fully compromised.

In conclusion, data from pharmacological interventions indicates that modulation of autophagic activity starting at early points of HFD administration is more successful in modifying the final levels of steatosis, clearly indicating a close relationship between autophagy and NAFLD development. However, when administration occurs at later points of the diet or even after it, results are more susceptible to variations and may be a result of differences in NAFLD development or in duration of the pharmacological intervention.

### Autophagic Genetic Interventions

In this section we analyzed all the articles found in our search that used genetic interventions to either stimulate or suppress autophagy in mouse livers and concomitantly measured hepatosteatosis outcome. The rundown of results obtained is presented in Tables 5–7 and divided into nutrient overload, fasting, or control steatosis models.

**TABLE 5:** Autophagy protein genetic interventions and obesity models.

References	Animal	Fat (% energy from fat), duration (weeks). Other information about diet	Genetic intervention	Autophagic target	Autophagy modulation (up/down)	Steatosis outcome (up/down)	Correlation (+/0/−)
Yang et al., 2010	*ob/ob*mice	regular chow diet	adenoviral-*Atg7* overexpression	ATG7	Up	Down	−
Kim et al., 2013	C57BL/6J	60%, 13	*Atg7* f/f; Alb-Cre mice (*Atg7*-Li KO)	ATG7	Down	Down	+
Byun et al., 2020	C57BL/6 + Ad-Jmjd3	60%, 8	adenoviral-*Atg7* shRNA	ATG7	Down	Up	−
Fernández et al., 2017	C57Bl6/129 Sv	42%, 8	*Atg4b*-null mice	ATG4B	Down	Up	−
Fernández et al., 2017	C57Bl6/129 Sv	30% sucrose	*Atg4b*-null mice	ATG4B	Down	Up	−
Xiong et al., 2012	C57BL/6J	60%, 12	adenoviral-*Atg14* overexpression	ATG14	Up	Down	−
Settembre et al., 2013	C57BL/6	42%, 12	TcfebloxP/loxP; Alb-Cre mice (*Tfeb*-Li KO)	TFEB	Down	Up	−
Settembre et al., 2013	C57BL/6	42%, 12	adenoviral-*TFEB* overexpression	TFEB	Up	Down	−
Zhang et al., 2018	C57BL/6J	60%, 16	adenoviral-*TFEB* overexpression	TFEB	Up	Down	−
Ma et al., 2013	C57BL/6J	60%, 5, fed	*Fip200* f/f; Alb-Cre mice (*Fip200*-Li KO)	FIP200	Down	Unchanged	0
Ma et al., 2013	C57BL/6J	60%, 5, fasted	*Fip200* f/f; Alb-Cre mice (*Fip200*-Li KO)	FIP200	Down	Down	+
Ma et al., 2013	C57BL/6J	60%, 5, fasted	*Fip200* f/f; adeno-Cre injection (*Fip200*- conditional KO)	FIP200	Down	Down	+
Li et al., 2014	C57BL/6J	45%, 12	Heterozygous *Ulk1*-KO mice (*Ulk1* +/−)	ULK1	Down	Up	−

*Articles using genetic interventions in the autophagy machinery and obesity models to promote steatosis, describing the diet fat content, intervention duration, liver steatosis, and LC3-II and p62 protein in levels in the liver in comparison to control groups (WT), normalized by a housekeeping protein. LC3-II and p62 were measured by the authors through SDS-PAGE Western blots and described semi-quantitatively or qualitatively. All animals were male mice. Positive correlation = steatosis is up, and autophagy is up OR steatosis is down, and autophagy is down; Negative correlation = steatosis is up, and autophagy is down OR steatosis is down, and autophagy is up.*

**TABLE 6:** Autophagy protein genetic interventions and fasting.

References	Animal	Fasting period (hours)	Genetic intervention	Autophagic target	Autophagy modulation (up/down)	Steatosis outcome (up/down)	Correlation (+/0/−)
Singh et al., 2009	C57BL/6	24	*Atg7* f/f; Alb-Cre mice (*Atg7*-Li KO)	ATG7	Down	Up	−
Shibata et al., 2009	not described	12, 24	*Atg7* f/f; Alb-Cre mice *(Atg7*-Li KO)	ATG7	Down	Down	+
Kim et al., 2013	C57BL/6J	24	*Atg7* f/f; Alb-Cre mice (*Atg7*-Li KO)	ATG7	Down	Down	+
Kwanten et al., 2014	C57BL/6J	24	*Atg7* f/f; Alb-Cre mice (*Atg7*-Li KO)	ATG7	Down	Down	+
Takahashi et al., 2020	C57BL/6	24	*Atg7* f/f; Alb-Cre mice (*Atg7*-Li KO)	ATG7	Down	Down	+
Saito et al., 2019	C57BL/6	not described	*Atg7* f/f; Alb-Cre mice (*Atg7*-Li KO)	ATG7	Down	Down	+
Takagi et al., 2016	C57BL/6J	36	*Atg5* f/f; Alb-Cre mice (*Atg5*-Li KO)	ATG5	Down	Down	+
Li et al., 2018	C57BL/6J	16	*Atg5* f/f; Alb-Cre mice (*Atg5*-Li KO)	ATG5	Down	Down	+
Takahashi et al., 2020	C57BL/6	6-48	*Atg5* f/f;Mx1-Cre (*Atg5*-Li conditional KO)	ATG5	Down	Down	+
Settembre et al., 2013	C57BL/6	24	TcfebloxP/loxP; Alb-Cre mice (*Tfeb*-Li KO)	TFEB	Down	Up	−
Ma et al., 2013	C57BL/6J	16	*Fip200* f/f; Alb-Cre mice (*Fip200*-Li KO)	FIP200	Down	Down	+

*Articles using genetic interventions in the autophagy machinery and fasting models to promote steatosis, describing fasting duration, liver steatosis, and LC3-II and p62 protein in levels in the liver in comparison to control groups (fed), normalized by a housekeeping protein. LC3-II and p62 levels were determined through SDS-PAGE Western blots in the manuscripts and described semi-quantitatively or qualitatively. All animals were male mice. Positive correlation = steatosis is up, and autophagy is up OR steatosis is down, and autophagy is down; Negative correlation = steatosis is up, and autophagy is down OR steatosis is down, and autophagy is up.*

**TABLE 7:** Autophagy protein genetic interventions plus chow or LFD diets.

References	Animal	Age at the end (weeks)	Genetic intervention	Autophagic target	Autophagy modulation (up/down)	Steatosis outcome (up/down)	Correlation (+/0/−)
Singh et al., 2009	C57BL/6	16	*Atg7* f/f; Alb-Cre mice (*Atg7*-Li KO)	ATG7	Down	Up	−
Yang et al., 2010	C57BL/6	14	adenoviral*-Atg7*shRNA	ATG7	Down	Up	−
Settembre et al., 2013	C57BL/6	not described	*Atg7* f/f; adeno-Alb-Cre injection (*Atg7*-Li conditional KO)	ATG7	Down	Up	−
Takahashi et al., 2020	C57BL/6	5	*Atg7* f/f; Alb-Cre mice (*Atg7*-Li KO)	ATG7	Down	Unchanged	0
Byun et al., 2020	C57BL/6 + Ad-Jmjd3	12	adenoviral-*Atg7*shRNA	ATG7	Down	Up	−
Fernández et al., 2017	C57Bl6/129 Sv	16	*Atg4b*-null mice	ATG4	Down	Unchanged	0
Xiong et al., 2012	C57BL/6J	12	adenoviral-Atg14 shRNA	ATG14	Down	Up	−
Ma et al., 2013	C57BL/6J	not described	Fip200 f/f; Alb-Cre mice (Fip200-Li KO)	FIP200	Down	Unchanged	0

*Articles using genetic interventions in the autophagy machinery to promote steatosis, describing liver steatosis, and LC3-II and p62 protein in levels in the liver in comparison to control groups (WT), normalized by a housekeeping protein. LC3-II and p62 levels were measured by the authors through SDS-PAGE Western blots and described semi-quantitatively or qualitatively. All animals were male mice. Positive correlation = steatosis is up, and autophagy is up OR steatosis is down, and autophagy is down; Negative correlation = steatosis is up, and autophagy is down OR steatosis is down, and autophagy is up.*

#### Nutritional Overload Models

Within nutrient overload steatosis models (Table 5), 13 different experiments were analyzed from 9 manuscripts which genetically modulated the expression of ATG7, ATG4B, ATG14, TFEB, FIP200, and ULK1. Five experiments (from 4 manuscripts) downregulated autophagic activity and observed increased steatosis outcome, while another 4 upregulated autophagic genes and observed decreased steatosis outcome. These were all assigned here as a negative correlation between autophagy and steatosis. In contrast, 3 other interventions (from 2 manuscripts) downregulated autophagic activity and showed a concomitant decrease in liver steatosis levels after diet, which were assigned as a positive correlation. Finally, one experiment downregulated autophagy and did not observe changes in steatosis after HFD.

Autophagy dysfunction in NAFLD models is well reported in the literature and is thought to be an important hallmark of steatosis worsening (Singh et al., 2009; Wang et al., 2018; Wu W. K. K. et al., 2018; Li et al., 2019b; Lian et al., 2020). Changes in autophagic flux are also observed in humans diagnosed with NAFL and NASH (González-Rodríguez et al., 2014) and prediabetic obese patients (Ezquerro et al., 2019). Autophagic degradation of LDs (lipophagy) is commonly thought necessary for TG turnover and FFA β-oxidation, which prevents lipotoxicity and further progression of the disease (Singh and Cuervo, 2011; Carotti et al., 2020). Thus, models with impaired autophagy activation are expected to accumulate more TGs in hepatocytes after an obesogenic diet, while activation of autophagy should prevent or improve this outcome. In agreement, autophagy-deficient *Atg4b*-null and *Tfeb*-Li KO mice exposed to different models of diet-induced steatosis presented increased hepatic TGs compared to WT (Settembre et al., 2013; Fernández et al., 2017). Also, experiments that overexpressed ATG7 in adult *ob/ob* mice (Yang et al., 2010), that overexpressed ATG14 in mice fed a HFD (Xiong et al., 2012), or that overexpressed TFEB in the livers of adult C57BL/6 mice before (Settembre et al., 2013) and even after 12 weeks of HFD (Zhang et al., 2018) all found decreased hepatic TG content, corroborating the idea that reactivation of autophagy in NAFLD can be a strategy to improve steatosis and other associated metabolic dysfunctions. In line with this, Byun et al. (2020) showed that adenoviral-mediated expression of the JMJD3 protein decreases hepatosteatosis induced by HFD in a mechanism dependent on ATG7 expression. This effect is abolished by *Atg7* KD, further indicating that autophagic activity counteracts lipid accumulation in NAFLD models (Byun et al., 2020).

While modulating ATG7 expression in adult obese mice showed a negative correlation with steatosis development, an experiment using mice born with depleted hepatic ATG7 showed the opposite effect. *Atg7* f/f; Alb-Cre (*Atg7*-Li KO) mice that were fed a HFD showed apparent lower accumulation of LDs in the liver (Kim et al., 2013). In agreement, early depletion of FIP200 in the liver of mice fed a HFD did not alter hepatic TG levels in animals in the fed state, even though it promoted impaired autophagic flux. In HFD-fed animals when fasted, early and late depletion of FIP200 decreased TG content compared to WT under the same conditions (Ma et al., 2013), suggesting that impaired autophagy can prevent lipid accumulation in certain contexts. Interestingly, FIP200 is part of an initiation complex in autophagy machinery, interacting with ULK1/2 to regulate the induction of autophagosome formation (Chen and Klionsky, 2011). However, the authors suggest that FIP200 may have other regulatory roles in autophagy, as they had an unexpected observation of accumulated LC3-II in KO animals, indicative of impaired late autophagy machinery rather than initiation. To support this, heterozygous *Ulk1*-KO mice (*Ulk1*±) fed a HFD showed opposite effects, increasing lipid accumulation in hepatocytes in comparison to WT animals (Li et al., 2014).

Although the majority of analyzed articles point to a protective role of autophagy in steatosis promoted by nutrient overload, there are conflicting data in the literature that cannot be overlooked. Comparing differences in experimental models and designs for each study can help unveil possible issues that may contribute toward this divergence. For instance, FIP200 and ATG7 are required for autophagy initiation and expansion phases meaning that Li-KO animals for these genes have virtually total hepatic autophagy impairment. These animals develop hepatomegaly with changes in hepatic structure (Komatsu et al., 2005; Kim et al., 2013; Ma et al., 2013), while showing decreased or unchanged accumulation of lipids upon dietary intervention. In contrast, *Atg4b*-null, *Tfeb*-Li KO, and *Atg7* KD by shRNA interventions, which were found to promote increased lipid accumulation, only partially compromise autophagy activation, which may be sufficient to keep basal autophagic functions in the liver. In fact, *Tfeb*-Li KO animals do not present significant liver histological changes compared to WT animals (Settembre et al., 2013). Therefore, time and type of genetic intervention as well as animal genetic background may contribute to apparent differences in literature data. Additionally, duration of diet stimulus may be a contributing factor. For example, Ma et al., 2013 did not observe changes in steatosis levels of *Fip200*-KO compared to WT animals after 5 weeks of diet without fasting stimulation. Since most of the articles ranged from 8 to 16 weeks of diet, the duration performed in this study may not be sufficient to observe the contribution of autophagy in hepatic steatosis, which is apparently more evident around 10 weeks, according to Zhang et al., 2018.

#### Fasting Models

Within fasting-induced steatosis models, we analyzed 11 experiments from 10 manuscripts that used genetic liver-specific KO models for ATG7, ATG5, TFEB, and FIP200 (Table 6). From these 11 experiments, 9 showed a positive correlation between autophagic activity and steatosis levels, meaning that KO animals showed lower TGs levels in the livers after fasting compared to WT. Two out of 11 experiments showed a negative correlation, where an increase in TG levels was observed in fasted KO animals.

When analyzing both experiments that showed a negative correlation, one evaluated the effects of ATG7 and the other of TFEB depletion. While results with *Atg7*-Li KO seem conflicting, TFEB genetic modulation experiments in the literature are apparently more consistent toward a protective role against steatosis. TFEB translocation to the nucleus is known to be necessary during fasting stimulus to promote the expression of genes related to lysosomal biogenesis, autophagic machinery and lysosome-autophagosome fusion (Settembre et al., 2013). Interestingly, TFEB activation by fasting may also increase lipid catabolism by upregulation of genes related to mitochondrial biogenesis and β-oxidation through transcriptional regulation of PGC1α (peroxisome proliferative activated receptor gamma coactivator 1 alpha), and PPARα (peroxisome proliferator activated receptor alpha). However, it was unclear if these effects were dependent or independent of canonical autophagy, since TFEB overexpression was not able to restore normal lipid levels in animals with ATG7 hepatic depletion in the fed condition. It is therefore possible that TFEB has a transcriptional regulatory role in lipid metabolism during fasting that differs from other autophagy-mediated regulations.

During prolonged starvation periods, autophagy is activated in hepatocytes, and is necessary for liver physiological adaptation (Takagi et al., 2016; Ruan et al., 2017; Ueno and Komatsu, 2017). LD numbers and size also increase as a strategy to avoid FFA lipotoxicity with higher lipid mobilization. However, the role for autophagy in fasting-induced steatosis remains ambiguous. LC3 was shown to associate with LDs during fasting. Two different roles can be attributed to this translocation. First, autophagosomes may form in LDs to engulf them, in order to promote the breakage of TGs by lysosomal lipases and release FFA for β-oxidation (lipophagy) (Singh et al., 2009). Second, autophagy machinery may be necessary for LD biogenesis during fasting (Shibata et al., 2009, 2010). Therefore, autophagy activation during fasting may have a double catabolic and anabolic regulatory participation in lipid metabolism.

In our search, 9 out of 10 experiments with genetic KO of proteins related to autophagy initiation (FIP200) and autophagosome expansion (ATG7 and ATG5) demonstrated lack of liver adaptation to fasting relative to lipid accumulation, favoring the anabolic role of autophagy. Different reasons may lead to reduced lipid contents in hepatocytes. FFA oxidation may be increased in autophagy-deficient animals, however, this is less likely since these animals probably accumulate damaged mitochondria due to impaired mitophagy, as has been reported in some models (Kwanten et al., 2014). Additionally, either TG or LD biosynthesis may be impaired, or liver export of lipids through VLDLs (very low-density lipoprotein) could be increased.

Different signaling pathways have been recently proposed to participate in this effect. Komatsu’s group published interesting data from two studies demonstrating that autophagy regulates the levels of NCoR1 (nuclear receptor co-repressor 1), a suppressor of LXRα (liver receptor X alpha) and PPARα transcription factors that, in turn, regulate the expression of genes related to lipogenesis and β-oxidation, respectively. Mice with impaired autophagy displayed NCoR1 accumulation and downregulation of genes associated with both TG synthesis and FFA oxidation. Thus, the authors suggest that autophagy-deficient models have hampered TG biosynthesis and overall lipid mobilization necessary for tissue adaptation to fasting (Saito et al., 2019; Takahashi et al., 2020). In fact, hepatocyte accumulation of lipids and FFA oxidation is necessary for ketogenesis increment during fasting (Rui, 2014), and both are compromised in autophagy-KO mice (Takagi et al., 2016). In agreement, Kim et al. (2013) also found that genes related to *de novo* FFA and TG synthesis were downregulated in *Atg7*-Li KO mice. The same study also found that KO mice had lower adipose tissue mass, which is the main source of lipids during fasting. Decreased fat mass was probably due to increased circulating FGF21, which promoted adipose tissue browning and increased systemic FFA oxidation. Therefore, endocrine changes in autophagy Li-KO mice may also contribute to the observed effects. Finally, another study suggested that impaired fasting-induced steatosis in *Atg5*-Li KO mice is not related to changes in *de novo* lipid biogenesis or β-oxidation levels, but rather to increased activation of NRF2 (nuclear factor erythroid 2–related factor 2), since double-knockout animals for ATG5 and NRF2 had restored phenotypes (Li et al., 2018).

Although results in the literature seem contradictory, clearly autophagy machinery is necessary for liver lipid metabolism adaptation to fasting. Mechanistically, the catabolic role of autophagy through degradation of LDs does not seem to be as evident in fasting-induced steatosis models as observed in nutrient-overload models, since almost all studies showed impaired accumulation of lipids in autophagy-deficient animals. Instead, the autophagic process appears to be part of a complex signaling network that may involve LD biogenesis and transcriptional regulation of metabolic pathways important to respond to physiological changes.

#### Control Models

In this section we analyzed the effects of autophagic genetic interventions in animals without any steatotic stimulation, to discuss the regulation of basal lipid metabolism by autophagy. We located 8 different experiments from 8 manuscripts, of which 4 showed negative correlation between autophagy modulation and steatosis outcome and 4 showed no alterations in steatosis (Table 7).

When analyzing the experiments targeting ATG7, we observe that similar genetic models showed divergent outcomes in the literature. For example, two different studies that infected mice with adenoviral-*Atg7* shRNA observed either increased or unchanged steatotic outcome in the basal physiological state (Yang et al., 2010; Byun et al., 2020). Similar data is present with *Atg7*-Li KO animals: one study shows higher hepatic lipid accumulation and another shows no alterations (Singh et al., 2009; Takahashi et al., 2020). Interestingly, we find that the duration of experiments may be an important contributing factor. For instance, Yang et al. (2010) analyzed the hepatic tissue at a shorter time (7–10 days) after Ad-*Atg7* shRNA infection compared to Byun et al. (2020) (4 weeks). The age of *Atg7*-Li KO mice at the end of the study was also lower in work by Takahashi et al. (2020) (5 weeks) compared to the study by Singh et al. (2009) (16 weeks). Therefore, we speculate that longer periods of ATG7 downregulation may be necessary to observe changes in hepatic lipid accumulation in the basal state. Although this might be relevant, several other features involving differences in genetic background and changes in environmental or experimental conditions may be equally responsible for literature divergence. In addition to the controversial results promoted by ATG7 modulation, other studies with depletion of ATG4B or FIP200 did not observe any changes in steatosis levels in the basal state compared to wild-type animals, while knockdown of ATG14 led to increased steatosis.

Overall, the effects of genetic modulation of autophagic genes on basal liver lipid metabolism are highly divergent in the literature. This suggests that the interplay between autophagy and liver lipid metabolism under normal physiological conditions is not a straightforward process. Instead, the interaction is context-dependent.

### Further Considerations

Over the past 10 years, different studies have been trying to elucidate the role of the autophagic process in lipid metabolism. Since then, many controversial ideas have emerged and are still being investigated. Autophagy machinery can apparently play different roles in hepatocyte lipid mobilization, regulating either catabolic or anabolic pathways, depending on context (Zechner et al., 2017). In our compilation of literature data, most studies indicated that inhibiting autophagy (pharmacologically or genetically) in NAFLD models increases steatosis. Likewise, promoting autophagic activity (pharmacologically or genetically), either during or after model development, improves steatosis outcome by decreasing lipid accumulation in hepatocytes. This is in line with the proposal that autophagic activity is necessary for lipid degradation during overnutrition-induced steatosis.

Interestingly, although there is still some conflicting data, this scenario is the opposite for fasting-induced steatosis models. In this context, most data using autophagy-deficient mice demonstrated an impaired capacity for lipid accumulation in response to fasting stimulus. Although it is tempting to hypothesize that autophagy’s role in steatosis development may vary according to the source of stimulation, it is possible that this apparent difference between NAFLD and fasting models found in our research comes from differences in the strategies used for autophagy modulation as well. For instance, studies investigating nutritional overload steatosis covered a greater variety of strategies, such as pharmacological inhibition/activation, genetic knockdown, knockout or overexpression of autophagic pathways. Conversely, all the results from the studies in the fasting group were from genetic knockout models. Additionally, only two studies investigated the effect of autophagy modulation on NAFLD and fasting steatosis within their work and both found similar outcomes, irrespective of the steatotic stimulus.

Even so, *in vitro* studies may help understand how the participation of autophagy in lipid metabolism can be modulated. For instance, inhibition of autophagy in hepatocytes and other mammalian cells treated with oleic acid leads to LD accumulation (Singh et al., 2009; Li et al., 2018), indicating that lipophagy is activated in this model. In contrast, autophagy is not necessary for LD degradation during acute amino acid starvation with HBSS in mammalian cells. Instead, autophagic degradation of organelles during HBSS starvation releases FFAs, that are converted to TGs in a protective mechanism dependent on DGAT1 activity, supporting LD formation (Rambold et al., 2015; Nguyen et al., 2017). Interestingly, lipophagy participation was observed during serum starvation, in media containing glucose and amino acids (Rambold et al., 2015). This suggests that lipophagy can be regulated by nutrient availability. In fact, different metabolites can regulate autophagy during fasting. Decreased amino acid levels after prolonged fasting inhibit mTORC1 activity and promote autophagy (Ueno and Komatsu, 2017). In contrast, fasting-induced increments in cytosolic acetyl-CoA derived from peroxisomal β-oxidation support mTORC1 activation, inhibiting autophagy/lipophagy (He et al., 2020). Curiously, recent data published as a pre-print suggests that mTORC1 activity may have differential roles in the regulation of autophagy and lipophagy, depending on its subcellular localization. The authors proposed that phosphorylation of Plin3 by mTORC1 at the surface of LDs is necessary for FIP200 and ATG16 recruitment to the organelle during oleic acid activation of lipophagy (Garcia-Macia et al., 2019). Based on this evidence, we speculate that amino acid availability may be necessary to support mTORC1 activity promoting autophagic machinery recruitment to LDs, allowing the participation of lipophagy in LD turnover. Conversely, lower amino acid concentrations during starvation, or even during prolonged fasting, may decrease lipophagy activation by similar mechanisms, while other lipolysis pathways can occur. New evidence on lipophagy mechanisms and its co-regulation with neutral lipolysis pathway are emerging fast (recently reviewed by Zechner et al., 2017; Schulze and McNiven, 2019; Shin, 2020), along with crosstalk signaling between autophagy and LDs (reviewed by Ogasawara et al., 2020), and will certainly help clarify current questions in the field.

The protective role of autophagy against steatosis development in NAFLD models is mainly associated with TG hydrolysis by lipophagy activation (Carotti et al., 2020). However, it is possible that autophagic activity may affect lipid metabolism directly or indirectly by different mechanisms. For instance, autophagy is necessary for mitochondrial quality control, and proper mitochondrial function is important for FA oxidation. In fact, mitophagy also counteracts NAFLD progression (Glick et al., 2012; Ma et al., 2013). Also, many genetic autophagy modulation models showed alterations in the expression of genes related to lipid biosynthesis and/or degradation (Yang et al., 2010; Kim et al., 2013; Ma et al., 2013), suggesting that transcriptional regulation of lipid metabolism by autophagic activity is also important. However, the mechanism supporting it is less clear in the context of diet-induced steatosis models compared to recent findings with fasting-induced steatosis (Saito et al., 2019; Takahashi et al., 2020). Finally, genetic autophagy modulation can promote changes in peripheral lipid metabolism even in liver-specific models (Kim et al., 2013; Settembre et al., 2013), which may also contribute toward hepatic steatosis levels. Taken together, these findings bring an additional layer, beyond lipophagy stimulation, to the discussion regarding the mechanism of autophagic activation in NAFLD models. Importantly, we highlight that although autophagic activation improved hepatic steatosis in most studies, chronic treatment with rapamycin may be detrimental to adipose tissue function and promote glucose intolerance (Houde et al., 2010; Paschoal et al., 2017; Wang Y. et al., 2017), which makes it a controversial NAFLD therapy.

Regarding the anabolic role of autophagy, there is still poor evidence if autophagic machinery can act directly on LD formation sites, but it is possible that proteins from the LC3/GABARAP family can play a role in the formation of this organelle, as for other ER-derived vesicles (Schaaf et al., 2016). Until now, different indirect mechanisms are proposed to explain autophagy participation in LD formation, involving transcriptional regulation of TG synthesis (Takahashi et al., 2020), regulation of NRF2 activity (Li et al., 2018), and endocrine regulation of FFA supply by the adipose tissue (Kim et al., 2013).

Recently, there is growing evidence of lysosome-dependent mechanisms of lipid metabolism regulation that do not involve autophagosome formation. Besides lipolysis facilitation by chaperon-mediated autophagy (Kaushik and Cuervo, 2015), lysosomes can form contact sites with LDs that mediate direct transfer of lipid content to the lysosome lumen, especially during nutrient deprivation conditions (Schulze et al., 2020). Additionally, a new SQSTM1-mediated autophagy-independent lysosomal degradation (SMAILD) pathway has been proposed to degrade SCAP (SREBF chaperone) proteins, necessary for the regulation of transcription factors related to lipid biosynthesis, while pharmacological activation of this pathway reduced hepatic steatosis (Zheng et al., 2020). These new discoveries corroborate the fact that a *Tfeb* Li-KO study showed different outcomes compared to other autophagic KO models during fasting-induced steatosis.

## Autophagy Markers

**FIGURE 3:**
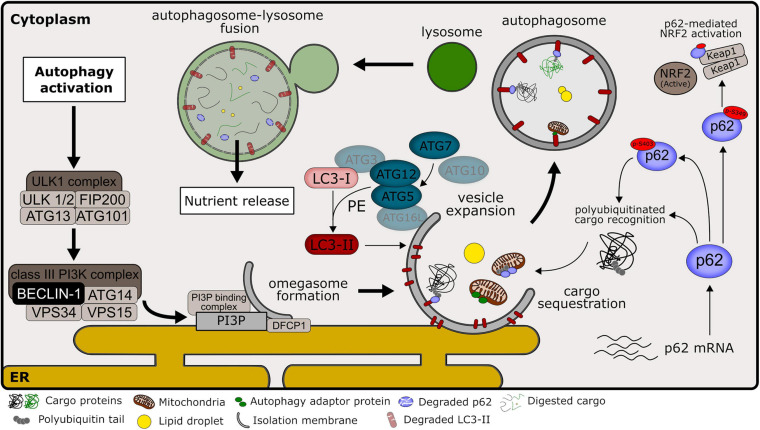
Overview of autophagy pathways in mammalian cells. Common autophagy markers and their participation in autophagic machinery, as discussed in Section “Autophagy Markers.” Signaling pathways that culminate in autophagy induction commonly lead to the activation of ULK1 kinase complex, promoting its localization at autophagy initiation sites near the endoplasmic reticulum (ER). ULK1 complex activity regulates the activation of the class III phosphatidylinositol 3-kinase (PI3K) complex, consisting of class III PI3K, VPS34, and other interacting proteins, including BECLIN-1. This complex is responsible for the production of phosphatidylinositol 3-phosphate (PI3P) at the site of early isolation membrane formation, which is essential for the nucleation step in the autophagic pathway. PI3P acts as a signaling molecule that recruits the double FYVE containing protein 1 (DFCP1) and other PI3P-binding proteins that promote the omegasome formation. At the expansion step, the autophagy-related protein 7 (ATG7) functions as an E1-like enzyme that catalyzes the conjugation of ATG12 to ATG5 in collaboration with the E2-like activity of ATG10. ATG12-ATG5 interacts with ATG16L at the vesicle membrane, marking the lipidation site. Microtubule-associated proteins 1A/1B light chain (LC3) at its mature form (LC3-I) are conjugated to phosphatidylethanolamine (PE) through the combined activity of ATG7 (E1-like enzyme), ATG3 (E2-like enzyme) and ATG12-ATG5 (E3 ligase enzyme). LC3 conjugated to PE (named LC3-II) is located at the autophagosome membrane and is important for the expansion and completion of the isolation membrane. P62/sequestosome-1 (named p62) works as an autophagy adaptor that recognizes polyubiquitinated cargos that will be sequestered for autophagic degradation due to their interaction with LC3-II. P62 phosphorylation at serine 403 (p-S403) increases affinity for polyubiquitinated targets and is promoted during autophagy activation. P62 phosphorylation at serine 349 (p-S349) is important for its competitive interaction with KEAP1 protein, promoting the activation of the NRF2 transcription factor, important during non-alcoholic liver disease (NALFD) development. Other autophagy adaptors besides p62 may participate during cargo recognition of selective autophagy. After autophagosome closure, the autophagic pathway proceeds with the formation of the autolysosome. The autophagosome external membrane fuses with the lysosome, releasing lysosome acidic hydrolases into the autophagosome lumen, and promoting cargo digestion. Importantly, LC3-II and p62 present within the autolysosome are also degraded in this process.

Autophagy is a very dynamic process within the cell. Moreover, the balance between autophagosome formation and degradation may quickly change in response to metabolic and environmental stimuli, adapting the flux to cellular necessities. This creates a challenge to correctly measure and interpret data that reflects changes in autophagic activity, especially in animal models. In our summary of literature results, most of the studies relied on the evaluation of LC3 forms and p62/SQSTM1 protein levels. To a lesser extent, changes in the levels of BECLIN-1 and ATG proteins were also measured in NAFLD models. Finally, a few studies also used electron microscopy or LC3 imaging (by immunofluorescence, IF or immunohistochemistry, IHC) as a method to measure autophagosome numbers. The participation of commonly used markers in the general autophagy pathway is illustrated in Figure 3. The overall summary of each marker and changes observed after steatosis is described in Table 8.

**TABLE 8:** Autophagy markers.

	LC3-II (WB)	LC3 II/I (WB)	LC3 imaging (IF or IHC)	p62	Beclin-1	Atg5	Atg7	Atg12	EM (autophagosome)
Up	30	20	14	72	12	7	5	2	10
Down	34	20	7	7	14	5	13	1	4
Unchanged	10	4	1	9	3	1	2	1	0
Total	74	44	22	88	29	13	20	4	14

*Markers and changes observed after steatosis is established.*

From all markers measured, p62 levels were the most consistent. From 88 studies, 72 found them increased in the livers of NAFLD models compared to control, while 16 found levels either decreased or unchanged. P62/SQSTM1 is most known as an adaptor that mediates the degradation of sequestered cargo through autophagy. It normally functions by binding to polyubiquitinated targets that in turn are delivered to autophagic digestion through p62 and LC3-II interaction. Thus, its levels are expected to correlate inversely with autophagic activity, since p62 itself is degraded within autophagolysosomes (Long et al., 2017). However, to evaluate p62 mRNA along with protein results is recommended in order to assist in data interpretation, once p62 can be intensively regulated at the transcriptional level (Klionsky et al., 2021). In the context of NAFLD, p62 is thought to accumulate in hepatocytes because of decreased autophagic flux. In severe cases, p62 can aggregate with insoluble protein inclusions known as Mallory-Denk bodies (MDBs). The observation of MDBs in hepatocytes is commonly associated with poor prognosis in NASH patients and correlates with proinflammatory M1-polarization of macrophages within liver biopsies (Zatloukal et al., 2007; Fukushima et al., 2018). Of note, a few articles opted to measure changes in insoluble and soluble fractions of p62, as an indication of protein aggregates (Park et al., 2014; Cho et al., 2018), which may be more indicated when studying NASH models. Additionally, other studies analyzed p62 phosphorylation levels at Ser403 as a marker, which is known to be an important step for the degradation of ubiquitinated proteins and protein aggregates (Matsumoto et al., 2011; Klionsky et al., 2021). Interestingly, p62 aggregation induced by lipotoxicity is dependent on its phosphorylation by TBK1 (TANK Binding kinase 1) at the same residue, while inhibition of this kinase prevented the formation of ubiquitin-p62 aggregates in mouse NASH models (Cho et al., 2018). In addition to its role in autophagic protein degradation, p62 has been proposed to participate in a Parkin-independent mitophagy mechanism that counteracts liver damage during NAFLD (Yamada et al., 2018). Regarding its role in lipid metabolism, it is not clear whether p62 acts directly as an adaptor that targets LDs to autophagic degradation, although there is data indicating it is present on the surface of the LDs and required for lipophagy (reviewed by Roberts and Olzmann, 2020). Finally, p62 is an important connector of autophagy and other cellular protective pathways. Its activity is known to promote NRF2 activation by competitively interacting with Keap1 through its KIR region and to promote its autophagic degradation, releasing NRF2 from the cytosol and allowing its nuclear translocation (Long et al., 2017). This mechanism is dependent on its phosphorylation at serine 349 (Ichimura et al., 2013; Sánchez-Martín et al., 2019). In turn, NRF2 itself is known to promote p62 expression, leading to a feedback loop against cellular stress (Jain et al., 2010; Taniguchi et al., 2016). Importantly, p62-dependent activation of NRF2 plays an important role against lipotoxicity in hepatocytes (Park et al., 2015; Lee et al., 2020). Moreover, accumulation of p62 due to impaired autophagic activity may lead to sustained activation of NRF2, which is a hallmark of many tumorigenic processes, including hepatocellular carcinoma (Taniguchi et al., 2016). Thus, the p62-NRF2 axis may be an important effector connecting impaired autophagy and NAFLD progression toward carcinoma.

Differently from p62 results, LC3-II levels presented significant variation. When compared to housekeeping proteins (mostly β-actin), we observed that 30 studies out of 74 found increased LC3-II levels, 34 found it decreased, and 10 found it unchanged. Similar proportions were obtained in studies that measured LC3-II in relation to LC3-I (LC3-II/I ratio). Importantly, many experts advise against LC3-II comparisons to LC3-I, mainly because immunoreactivity tends to be different between both forms, thus their variations in blots are therefore not proportional, which may complicate result interpretation (Mizushima and Yoshimori, 2007; Bonam et al., 2020; Klionsky et al., 2021). While LC3-II levels correlate with autophagosome numbers in the cell, changes observed in this marker cannot support conclusions regarding the intensity of autophagic activity if analyzed alone, because autophagosome levels in the cell are a sum of formation and degradation rates, and LC3-II changes can indicate variations in both processes. This is exemplified by the fact that about half of the studies found it either increased or decreased in NAFLD models, while most reached the conclusion of impaired autophagic activity. To that end, many studies measured autophagic flux *in vivo* by comparing animals that were injected with lysosome inhibitors a few hours before sacrifice to untreated animals within the same group. In addition, multiple methodological strategies may be used in parallel to complement the limitations of a single analysis or marker, allowing better interpretation of changes in autophagy dynamics *in vivo* (Bonam et al., 2020; Klionsky et al., 2021). Importantly, autophagic flux can vary greatly in the hepatic tissue in physiological states with circadian cycles and periods after feeding (Ma et al., 2011; Martinez-Lopez et al., 2017; Toledo et al., 2018). Thus, control for these variables is an important concern during *in vivo* analysis of autophagy and may also be a source of variability within and between studies.

Similar to LC3-II findings, other markers related to autophagy initiation and autophagosome formation machinery also presented increased variability. For example, BECLIN-1 levels were found increased in 12 out of 29 studies, decreased in 14 and unchanged in 3. BECLIN-1 is a protein part of the VPS34 complex that functions downstream of ULK-1 in the autophagy initiation cascade. Its phosphorylation is known to be necessary to activate VPS34 and VPS15 kinase activity, increasing local production of phosphatidylinositol-3-phosphate and inducing the formation and expansion of the phagophore (Zachari and Ganley, 2017). Although autophagy can be initiated through BECLIN-1-independent mechanisms (Klionsky et al., 2021), after the induction of phagophore formation, membrane expansion and completion steps require LC3-I conjugation to phosphatidylethanolamine (LC3-II), a process that is dependent on the ATG5-ATG12 complex and ATG7 activity (reviewed by Chen and Klionsky, 2011; Ueno and Komatsu, 2017; Clarke and Simon, 2019). Thus, measuring the levels of these proteins can be indicative of the capacity to induce autophagosome formation and to activate autophagy. Interestingly, we observed that alterations in the levels of BECLIN-1 and ATG7 correlated with alterations in LC3-II levels in the studies that analyzed both markers concomitantly, as would be expected. All studies that found decreased BECLIN-1 or decreased ATG7 also found lower LC3-II levels (or LC3-II/I ratios). Conversely, 3 out of 4 and 9 out of 11 studies that found increased ATG7 or BECLIN-1 also found increased LC3-II, respectively. Mechanistically, the expression of these proteins can be regulated by important transcription factors that act in a manner sensitive to metabolic states in the hepatic tissue, such as TFEB and PPARα (Lee et al., 2014; Napolitano and Ballabio, 2016). In addition, protease activity may also have a contribution, as has been shown for calpain-mediated degradation of ATG7 in obesity (Yang et al., 2010).

Due to the specificity of each protein in the autophagic signaling cascade, we highlight the fundamental necessity to evaluate different markers simultaneously to reach better conclusions regarding alterations in autophagic activity. To that end, many methods to measure autophagy during *in vivo* experiments are being developed and will certainly contribute to improving our knowledge.

## Author Contributions

VR and PK performed conceptualization and data curation. VR, PK, and AK contributed to funding and writing. All authors contributed to the article and approved the submitted version.

## Conflict of Interest

The authors declare that the research was conducted in the absence of any commercial or financial relationships that could be construed as a potential conflict of interest.
