# Supplementary material for: Autophagy in Hepatic Steatosis: A Structured Review
Source: Front Cell Dev Biol. 2021 Apr 15;9:657389. doi: 10.3389/fcell.2021.657389 (PMC8081956; doi:10.3389/fcell.2021.657389)
Supplement: Supplementary file 1 [file Table_1.DOCX]

## Table S1: Experiments with high fat diets assessing LC3-II and p62 levels (data in Figure 1)

Articles describing diet fat content (percentage of energy), duration of the intervention, and LC3-II and p62 protein in levels in the liver in comparison to control groups (chow or LFD), normalized by a housekeeping protein. LC3-II and p62 levels were obtained by the authors through SDS-PAGE Western blots and described semi-quantitatively or qualitatively. All experiments observed increases lipid accumulation in the liver. (N/A = not available)

| Reference | Species, strain, sex | Fat (% energy from fat), duration (weeks)  Other information about diet | LC3-II | p62 |
| --- | --- | --- | --- | --- |
|  |  |  | (vs. LFD or Chow) | |
| (Mei et al., 2011) | Mice, C57BL/6, Male | 40, 12  0.15% cholesterol | Up | N/A |
| (Sharma et al., 2011) | Mice, C57BL/6, Male | 45, 8 | Unchanged | N/A |
| (Li et al., 2013) | Mice, C57BL/6, Male | 49, 24 | Down | N/A |
| (Lin et al., 2013) | Mice, C57BL/6, Male | 60, 12  9.4% sucrose | Up | N/A |
| (Tan et al., 2013) | Mice, C57BL/6, Male | 23, 8  0.19% cholesterol, 42.7% sucrose | Up | Unchanged |
| (González-Rodríguez et al., 2014) | Mice, C57BL/6, Male | 42, 30  0.2% cholesterol, 34.1% fructose | Up | Up |
| (Park et al., 2014) | Mice, C57BL/6, Male | 60, 8  14.6% sucrose | Up | Up |
| (Sinha et al., 2014) | Mice, C57BL/6, Male | 60, 8  9.4% sucrose | Unchanged | N/A |
| (Xiao et al., 2014) | Rat, Sprague-Dawley, Female | 30, 12 | Up | Down |
| (López-Vicario et al., 2015) | Mice, C57BL/6, Male | 60, 16 | Down | Unchanged |
| (Machado et al., 2015) | Mice, C57BL/6, Male | 45, 16  0.2% cholesterol, 21.1% fructose | Unchanged | N/A |
| (Zeng et al., 2015) | Mice, C57BL/6, Male | 60, 12  9.4% sucrose | Down | N/A |
| (Zhang et al., 2015b) | Mice, 129/SvJ, Male | 60, 4 | Unchanged | Unchanged |
| (Ezquerro et al., 2016) | Rat, Wistar, Male | 60, 16  14.6% sucrose | Unchanged | Unchanged |
| (He et al., 2016a) | Mice, C57BL/6, Male | 60, 12  9.4% sucrose | Down | Up |
| (He et al., 2016b) | Mice, C57BL/6, Male | 60, 12  9.4% sucrose | Down | Up |
| (Hsu et al., 2016) | Mice, C57BL/6, Male | 45, 8  0.0197% cholesterol, 20.09% sucrose | Up | N/A |
| (Shen et al., 2016) | Mice, C57BL/6, Male | 60, 16  9.4% sucrose | N/A | Down |
| (Tanaka et al., 2016) | Mice, C57BL/6, Male | 60, 8  6.75% sucrose | Up | Up |
| (Tanaka et al., 2016) | Mice, C57BL/6, Male | 60, 16  6.75% sucrose | Up | Up |
| (Tong et al., 2016) | Mice, C57BL/6, Male | 60, 16  9.4% sucrose | N/A | Up |
| (Deng et al., 2017) | Mice, C57BL/6, Male | 60, 19  9.4% sucrose | N/A | Up |
| (Ding et al., 2017) | Rat, Wistar, Male | 41.26, 18 | Down | Unchanged |
| (Guo et al., 2017) | Mice, C57BL/6, Male | 45, 20  20.6% sucrose | Down | Up |
| (Hsiao et al., 2017) | Mice, C57BL/6, Male | 53, 8  15% sucrose | Unchanged | N/A |
| (Iannucci et al., 2017) | Rat, Wistar, Male | 50, 1 | Unchanged | N/A |
| (Rosa-Caldwell et al., 2017) | Mice, C57BL/6, Male | 42, 8  0.15% cholesterol, 35% fructose | Down | Unchanged |
| (Wang et al., 2017a) | Mice, C57BL/6, Male | 58, 24 | N/A | Up |
| (Wang et al., 2017b) | Mice, Kumming, Male | 60, 12  6.75% sucrose | Down | Up |
| (Wang et al., 2017d) | Mice, C57BL/6, Male | 42, 11  0.2% cholesterol, 34.1% fructose | Down | Up |
| (Wang et al., 2017c) | Mice, C57BL/6, Male | 60, 16, MCFA enriched  9.4% sucrose | Up | Unchanged |
| (Wang et al., 2017c) | Mice, C57BL/6, Male | 60, 16,  LCFA enriched  9.4% sucrose | Up | Up |
| (Liu et al., 2018a) | Rat, Sprague-Dawley, Male | 60, 8  9.4% sucrose | N/A | Up |
| (Liu et al., 2018b) | Mice, C57BL/6, Male | 60, 10 | Down | Up |
| (Piacentini et al., 2018) | Mice, C57BL/6, Male | 42, 16  0.2% cholesterol, 34.1% sucrose | N/A | Down |
| (Porcu et al., 2018) | Mice, C57BL/6, Male | 42, 16  0.2% cholesterol, 34.1% fructose | N/A | Down |
| (Porcu et al., 2018) | Mice, C57BL/6, Female | 42, 16  0.2% cholesterol, 34.1% fructose | N/A | Down |
| (Qian et al., 2018) | Mice, C57BL/6, Male | 60, 16 | Down | Up |
| (Tan et al., 2018) | Mice, C57BL/6, Female | 60, 6  9.4% sucrose | N/A | Up |
| (Zhang et al., 2018b) | Mice, C57BL/6, Male | 60, 8  9.4% sucrose | N/A | Unchanged |
| (Chen et al., 2019a) | Mice, C57BL/6, Male | 42, 12 | N/A | Unchanged |
| (Cheng et al., 2019) | Mice, C57BL/6, Male | 42, 16  0.2% cholesterol, 34% sucrose | N/A | Up |
| (la Fuente et al., 2019) | Mice, C57BL/6, Male | 60, 20  9.4% sucrose | Up | Unchanged |
| (Ohashi et al., 2019) | Mice, BALB/c, Male | 60, 9 | Down | Up |
| (Qian et al., 2019) | Mice, C57BL/6, Male | 60, 10  9.4% sucrose | N/A | Up |
| (Ren et al., 2019) | Mice, C57BL/6, Male | 60, 12  9.4% sucrose | Down | Up |
| (Shi et al., 2019a) | Mice, C57BL/6, Male | 42, 8  0.2% cholesterol, 34% sucrose | Down | N/A |
| (Shi et al., 2019b) | Mice, C57BL/6, Male | 60, 25 | N/A | Unchanged |
| (Wang et al., 2019a) | Mice, C57BL/6, Male | 60, 18 | Unchanged | Up |
| (Wang et al., 2019b) | Mice, C57BL/6, Male | 60, 16 | Up | Up |
| (Zhu et al., 2019) | Mice, C57BL/6, Male | 60, 168 | N/A | Up |
| (Cañadas-Lozano et al., 2020) | Mice, C57BL/6, Male | 45, 84 | N/A | Down |
| (Lee et al., 2020) | Mice, C57BL/6, Male | 60, 13 | N/A | Down |
| (Liu et al., 2020) | Mice, C57BL/6, Male | 60, 12 | Down | Up |
| (Niture et al., 2020) | Mice, C57BL/6, Male | 45, 16 | Unchanged | N/A |
| (Tang et al., 2020) | Mice, C57BL/6, Male | 60, 16  8.8% sucrose | N/A | Up |
| (Wu et al., 2020) | Mice, C57BL/6, Male | 40, 12  0.15% cholesterol, 34% fructose | Up | Up |
| (Xue et al., 2020) | Mice, C57BL/6, Male | 42, 8  0.2% cholesterol, 34% sucrose | Down | Up |
| (Zhang et al., 2019) | Mice, C57BL/6, Male | 60, 18 | N/A | Up |
