# Supplementary material for: Autophagy in Hepatic Steatosis: A Structured Review
Source: Front Cell Dev Biol. 2021 Apr 15;9:657389. doi: 10.3389/fcell.2021.657389 (PMC8081956; doi:10.3389/fcell.2021.657389)
Supplement: Supplementary file 2 [file Table_2.DOCX]

## Table S2: Experiments with HFDs and genetic models

Articles describing diet fat content (percentage of energy), duration of the intervention, and LC3-II and p62 protein in levels in the liver in comparison to control groups (chow or LFD), normalized by a housekeeping protein. LC3-II and p62 levels were obtained by the authors through SDS-PAGE Western blots and described semi-quantitatively or qualitatively. All animals were male C57BL/6 mice. (N/A = not available)

| Reference | Genetic intervention | Fat (% energy from fat), duration (weeks)  Other information about diet | Steatosis | LC3-II | p62 |
| --- | --- | --- | --- | --- | --- |
|  |  |  | (vs. chow or LFD) | | |
| (Tan et al., 2013) | Hfe KO | 23, 8  0.19% cholesterol, 42.7% sucrose | Up | Unchanged | Up |
| (Li et al., 2013) | *Trpv1* knockout | 49, 24 | Up | Down | N/A |
| (Liu et al., 2015b) | ob/ob | 60, 10 | Up | Up | N/A |
| (López-Vicario et al., 2015) | *fat-1* | 60, 16 | Up | Unchanged | Unchanged |
| (Liu et al., 2015a) | *ApoE* KO | 37, 24  1.25% cholesterol, 1.16% fructose | Up | Unchanged | Unchanged |
| (Deng et al., 2017) | S1P KD | 60, 19  9.4% sucrose | Unchanged | N/A | Unchanged |
| (Deng et al., 2017) | S2P KD | 60, 19  9.4% sucrose | Unchanged | N/A | Unchanged |
| (Deng et al., 2017) | S1P and S2P KD | 60, 19  9.4% sucrose | Unchanged | N/A | Unchanged |
| (Guo et al., 2017) | APN KO | 45, 20  20.6% sucrose | Up | Down | Unchanged |
| (Piacentini et al., 2018) | Transglutaminase 2 KO | 42, 16  0.2% cholesterol, 34.1% sucrose | Up | N/A | Unchanged |
| (Qian et al., 2018) | *Gsnor* KO | 60, 16 | Up | Unchanged | Unchanged |
| (Lee et al., 2018a) | *Ldlr* KO | 45, 24  0.2% cholesterol, 34.1% fructose | Up | Up | Unchanged |
| (Qian et al., 2019) | *Inos* KO | 60, 10  9.4% sucrose, fed | Unchanged | N/A | Down |
| (Wu et al., 2020) | *Mlkl* −/− KO | 40, 12  0.15% cholesterol, 34% fructose | Unchanged | Unchanged | Unchanged |
